# Morning blood pressure surge and its relation to insulin resistance in patients of reproductive age with polycystic ovary syndrome

**DOI:** 10.1186/s12958-018-0394-2

**Published:** 2018-08-09

**Authors:** Hasan Kadi, Eyup Avci, Akin Usta, Abdullah Orhan Demirtaş

**Affiliations:** 1 Cardiology Department, Balikesir University Faculty of Medicine, Balikesir, Turkey; 2 Gynecology and Obstetrics Department, Balikesir University Faculty of Medicine, Balikesir, Turkey

**Keywords:** Morning blood pressure surge, Insulin resistance, Polycystic ovary syndrome

## Abstract

**Background:**

Our aim in this study was to investigate morning blood pressure surge (MBPS) in patients of reproductive age with polycystic ovary syndrome (PCOS) and its relation to insulin resistance (IR).

**Methods:**

Fifty-three patients with PCOS without additional illness were included in the study. Forty-two age-matched subjects without PCOS were selected as the control group. All study subjects underwent 24-h blood pressure monitoring. Patients with additional illnesses, drug users, smokers, and alcohol and drug abusers were excluded. Blood insulin, fasting glucose, lipid profile, and hormone profile were measured. Insulin resistance was calculated using the HOMA-IR formula.

**Results:**

Median age (years) was 27 (20–33) in the PCOS group and 27 (22–33) in the control group. Body mass index was higher in the PCOS group. Office systolic and diastolic blood pressure was higher in the PCOS group. Mean awakening 2-h BPs (mmHg) was 110 ± 7 in the control group and 118 ± 5 in the PCOS group (*p* < 0.001). Mean MBPS (mmHg) was 21 ± 6 in the control group and 29 ± 8 in the PCOS group. Mean MBPS was higher in the PCOS group (*p* < 0.001). IR was more frequent in the PCOS group. Based on logistic regression analysis, the presence of PCOS and IR were independent predictors for MBPS.

**Conclusions:**

The results of our study showed that MBPS increased excessively when compared to non-PCOS controls in young women with PCOS during reproductive age. In addition, PCOS and insulin resistance were independent risk factors for exaggerated MBPS.

## Background

Polycystic ovary syndrome (PCOS) is one of the most common endocrine disorders in women during reproductive age and is an important cause of infertility in women [1, 2]. Although the frequency of the disease varies according to the method used for diagnosis and the population studied, it has been reported to be approximately 5–10% of women of reproductive age [3]. PCOS is associated with undesired metabolic profiles and cardiovascular outcomes, and insulin resistance is frequently encountered in these patients [4, 5]. In a very recent study, insulin resistance in patients with PCOS was similar to that in prediabetic patients [6]. Insulin resistance plays a key role in metabolic and reproductive abnormalities in patients with PCOS [7].

Blood pressure has diurnal variation and shows a marked increase upon morning awakening when compared to the low levels seen during the night. The difference between the mean systolic blood pressure in the first two hours after awakening and the lowest blood pressure at night is defined as morning blood pressure surge (MBPS) [8]. It has been known for a long time that there is a relationship between blood pressure, which is high in the mornings, and cardiovascular events in the morning. In addition, exaggerated MBPS has also been shown to be an independent risk factor for adverse cardiovascular events [9, 10]. Our aim in this study was to investigate MBPS in patients of reproductive age with PCOS and determine its relation to insulin resistance.

## Methods

### Patient group

In the period between February 2017 and August 2017, outpatients who were diagnosed with PCOS for first-time in the Department of Obstetrics and Gynecology at the Medical Faculty of Balikesir University were included in the study. The PCOS prevalence in our clinic is 8%.

### Control group

In the obstetrics and gynecology outpatient clinic, a control group of individuals was evaluated, and PCOS was not detected in the period between February 2017 and August 2017. Exercise stress tests and echocardiography were performed to exclude patients with cardiovascular disease from the control group and the PCOS group. Informed written consent was obtained from all study subjects. The study protocol was approved by the institutional ethics committee.

### Exclusion criteria

Patients receiving treatment for PCOS were not taken into the study. Patients using antihypertensive or psychotropic drugs were excluded from the study. Patients were also excluded if they had known hypertension, masked hypertension, past CVO, known cardiovascular disease, diabetes, cardiovascular drug use, hormonal treatment for PCOS, significant systemic disease, pregnancy, atrial fibrillation, major arrhythmia, sleep apnea syndrome, morbid obesity (BMI > 40 kg/m^2^), autonomic dysfunction, chronic kidney disease, or diabetic neuropathy. Psychiatric patients and those using alcohol, tobacco, or drugs were further excluded.

### Office blood pressure measurements

After a minimum of five minutes of rest, the average of two different measurements taken five-minutes apart was recorded for each subject.

### Ambulatory blood pressure monitoring

An automated ambulatory blood pressure monitoring (ABPM) device was used as a 24-h blood pressure monitor (Mobil-Q-Graph, Germany). The cuff was placed on the nondominant arm. If there was greater than a five mmHg difference between the initial measurement of the BPP and the first measurement of the ABPM device, the cuff was removed and reconnected and the measurement repeated. If there was a difference greater than five mmHg between the first and second measurements, that patient was not taken into the study. The ABPM was set to measure at 15-min intervals during daytime hours and at 30-min intervals during nighttime hours. The patients were told to remain immobile during the measurement and to keep the arm at heart level. Patients were asked to record their sleeping and morning wake-up times. If there was less than two hours between the wake-up time in the morning and the last measurement, the patient was left out of work. Erroneous and incorrect readings were deleted. If more than 20% of all measurements were inappropriate, the patient was not taken to work. Day and night averages and morning surge calculations were calculated based on the individual sleeping and departure times of the patients.

### Blood sampling

In all patients and control group subjects, follicle-stimulating hormone (FSH), luteinizing hormone (LH), dehydroepiandrosterone sulfate (DHEAS), fasting blood glucose, triglyceride (TG), cholesterol (TG), thyroid hormone (TSH), prolactin, low-density lipoprotein-cholesterol (LDL-C), high-density lipoprotein-cholesterol (HDL-C), C-reactive protein (CRP), insulin, free testosterone, and 17-hydroxyprogesterone were measured. A comprehensive ultrasonography (USG) study of all patients and control group subjects was performed by experienced ultrasonographers.

### The homeostasis model assessment of insulin resistance (HOMA-IR)

HOMA-IR was calculated according to the following formula: HOMA-IR = fasting plasma insulin (mU/ml) x fasting plasma glucose (mmol/l)/22.5 [11]. If the calculated HOMA-IR was greater than 2.5, the patient was considered to have insulin resistance.

#### Definitions

##### Definition of PCOS

The diagnosis of PCOS was determined according to the Rotterdam ESHRE/ASRM–Sponsored PCOS Consensus Workshop Group Revised 2003 consensus on diagnostic criteria and long-term health risks related to polycystic ovary syndrome (PCOS) recommendations [12].

##### Definition of morning blood pressure surge

Following the method used by Kario and colleagues [8], MBPS was calculated by subtracting the lowest systolic blood pressure at night (the average BP of 3 readings centered on the lowest night-time measurements) from mean systolic BP in the first two hours after awakening.

#### Statistical analyses

The statistical software package SPSS for Windows 20.0 (SPSS Inc. Chicago, IL, USA) was used for the statistical analysis. Continuous data that fit a normal distribution are shown as mean ± standard deviation, and those that do not fit a normal distribution are presented as median values (minimum and maximum values). Continuous data were compared using t-tests or Mann-Whitney U test. Categorical variables were expressed as frequencies and percentages. Comparisons between categorical variables were done using a chi-square test. Logistic regression analyses were performed by accepting primary outcome measures as (mean MBPS was used as the cut-off level) dependent variable. Insulin resistance, presence of PCOS, and body mass index were independent variables in these analyses. A *p* value of less than 0.05 was considered significant.

## Results

Fifty-three patients with PCOS were included in the study. Forty-two age-matched subjects without PCOS were selected as the control group. Median age (years) was 27 (20–33) in the PCOS group and 27 (22–33) in the control group. Median body mass index (kg/m^2^) was 22 (19–28) in the control group and 24.1 (19.7–29.5) in the PCOS group. Body mass index was higher in the PCOS group (*p* = 0.001). Creatinine levels were similar between groups. Plasma insulin levels, HOMA-IR, office systolic and diastolic blood pressure, total cholesterol, triglyceride, LDL-C, HDL-C, and presence of insulin resistance were statistically different between groups. Basal characteristics and clinic and laboratory findings of the groups are shown in Table 1. Mean awakening two-hour BP (mmHg) was 110 ± 7 in the control group and 118 ± 5 in the PCOS group. Mean awakening two-hour BP was higher in the PCOS group (*p* < 0.001). Mean MBPS (mmHg) was 21 ± 6 in the control group and 29 ± 8 in the PCOS group. Mean MBPS was higher in the PCOS group (*p* < 0.001). The ABPM results of the groups are presented in Table 2.

**Table 1:** Basal characteristics of the groups

Variable\Group	Control Group	PCOS Group	*p*
Age, year	27 (20–33)	27.5 (22–33)	0.383
BMI, kg/m^2^	22 (19–28)	24.1 (19.6–29.5)	0.001*
Insulin, pg/ml	8 (3.38–45.56)	18.9 (4.85–48.6)	< 0.001*
Office systolic BP, mmHg	110.5 ± 9.5	120.5 ± 7.2	< 0.001*
Office diastolic BP, mmHg	72.6 ± 6.2	73.4 ± 5.4	0.478
HOMA-IR	1.75 (0.68–9.54)	4.27 (1.04–9.36)	< 0.001*
Creatinine, mg/dl	0.7 (0.6–0.9)	0.8 (0.6–0.9)	0.135
Total Cholesterol, mg/dl	169 ± 24	175 ± 23	0.224
Triglyceride, mg/dl	123 (81–210)	156 (68–247)	0.044*
HDL-C, mg/dl	52 (34–65)	45.5 (33–66)	0.004*
LDL-C, mg/dl	90 ± 30	100 ± 20	0.044*
Insulin resistance, *n* (%)	22 (30)	29 (76)	< 0.001*

*BP* Blood pressure, *BMI* Body mass index, *HOMA-IR* The homeostasis model assessment of insulin resistance, *HDL-C* High density lipoprotein cholesterol, *LDL-C* Low density lipoprotein cholesterol, *PCOS* Polycystic ovary syndrome. Age, insulin, HOMA-IR, creatinine, and triglyceride were presented as median (maximum-minimum), total cholesterol, LDL-C, were presented as mean standard ± deviation. * indicated a significant difference between controls and patients with polycystic ovary syndrome

**Table 2:** Ambulatory blood pressure monitoring results of the groups

Variable\Group	Control Group	PCOS Group	*p*
Day-time BPs	110 ± 7.6	111 ± 7.4	0.695
Day-time BPd	67.5 ± 6.8	69.7 ± 5.3	0.113
Night-time BP	102 ± 7.7	106 ± 6.3	0.015*
Night-time BPd,	61 ± 5.2	62 ± 4.6	0.197
Night-time lovest BPs	91.3 ± 5.9	87 ± 4.5	< 0.001*
Post awakening 2 h BPs	111 ± 6.3	121 ± 3.6	< 0.001*
24 h BPs	110 ± 6.3	111 ± 5.9	0.428
24 h BPd	66 ± 5	68 ± 5.3	0.175
BP surge	19.74 ± 4.6	33.7 ± 5.5	< 0.001*

BPs: Systolic blood pressure, BPd: Diastolic blood pressure

Blood pressure measurements and calculations were presented as mmHg and mean ± standard deviation. * indicated a significant difference between controls and patients with polycystic ovary syndrome

In the logistic regression analysis, categorized MBPS were accepted as dependent variables and body mass index, presence of PCOS, and insulin resistance were defined as independent variables. Based on logistic regression analysis, the presence of PCOS and IR are independent predictors for MBPS. Results of logistic regression analysis are presented in Table 3.

**Table 3:** Results of multivariate logistic regression analysis for dependent variable morning blood pressure surge

Variable	*P*	OR	95% CI
PCOS	0.016*	3.8	1.28–11.2
IR	0.045*	3.1	1.02–9.34
BMI	0.091*	1.2	0.97–1.43

*BMI* Body mass index, *CI* Confidence interval, *IR* İnsulin resistance, *OR* Odds ratio, *PCOS* Polycistic ovary syndrome. * indicated a significant difference between controls and patients with polycystic ovary syndrome

## Discussion

The cause of PCOS is not fully known. However, as with most complicated diseases, both environmental and genetic factors are influential in the pathogenesis of the disease. There are many studies showing that insulin resistance plays an important role in the pathogenesis of PCOS. Patients with PCOS have a higher frequency of impaired fasting glucose [13], higher HOMA-IR levels as an indicator of insulin resistance in peripheral tissues [14], and a higher incidence of diabetes mellitus [15]. In our study, HOMA-IR and insulin resistance were significantly higher in patients with PCOS than in controls. It has been reported that patients with PCOS have deteriorated metabolic profiles, increased blood triglyceride and cholesterol levels, and increased frequency of metabolic syndrome [16, 17]. In our study, compared with controls the PCOS group had higher cholesterol and LDL-C levels and lower HDL-C levels consistent with the literature.

Blood pressure disorders and hypertension are common in patients with PCOS. However, the relationship between hypertension and PCOS has not been fully clarified. In some studies, the frequency of hypertension was higher in women with PCOS than in women without PCOS [18, 19], and in some studies, it was reported that the frequency of hypertension did not differ in non-PCOS women, but the synergistic effect of excess weight gain with PCOS led to a rise in blood pressure [20, 21].

In our study, office blood pressure measurements were higher in the PCOS patient group compared to the control group, although not at hypertensive levels. However, when ABPM results were analyzed, daytime and nighttime mean systolic and diastolic blood pressures did not differ between groups. The fact that our study group was composed of young subjects and that the groups were similar in terms of body mass index may be the cause of such a result.

MBPS refers to the fluctuation in blood pressure from the night to the morning, and ambulatory blood pressure was measured using a monitor. In many studies, a close association between MBPS and cerebrovascular and cardiovascular diseases has been established. Kario et al. [8] demonstrated that elderly patients with exaggerated MBPS are at greater risk of cerebrovascular disease. In addition, exaggerated MBPS has been shown to be associated with future cardiovascular adverse events in both normotensives and well-controlled hypertensives [22]. It is also reported that exaggerated MBPS increases cardiovascular mortality [23] and prognostic value in cardiovascular disease [24]. It has been suggested that the underlying mechanism of exaggerated MBPS may be explained by insulin resistance, increased sympathetic activity, and increased renin-angiotensin-aldosterone system activity. In our study, both PCOS and insulin resistance were independent risk factors for exaggerated MBPS as demonstrated by the logistic regression analysis. These results indicate a close interaction between exaggerated MBPS, insulin resistance, and PCOS.

### Study limitation

The most important limitation of our study was the relatively small number of patients. Our study has elucidated some of the possible mechanisms by proving that the cause of exaggerated MBPS is insulin resistance. In addition, further studies should be undertaken to examine the possible contributions of cardiovascular risk factors which are frequently associated with PCOS. The effects of the sympathetic nervous system and the renin-angiotensin-aldosterone system should be investigated, and their possible effects on MBPS should be demonstrated.

## Conclusion

The results of our study show that, when compared to non-PCOS controls, MBPS increased excessively in young women with PCOS during reproductive age. In addition, PCOS and insulin resistance are independent risk factors for exaggerated MBPS. These results show that increased MBPS in patients with PCOS can be explained by insulin resistance. Thus, measures to decrease insulin resistance (lifestyle changes, dietary recommendations and physical exercise) may have to be considered earlier to decrease the potential risks of exaggerated MBPS in patients with PCOS in this age group.
